# CSF Biomarkers in the Early Diagnosis of Mild Cognitive Impairment and Alzheimer’s Disease

**DOI:** 10.3390/ijms24108976

**Published:** 2023-05-19

**Authors:** Vasileios Papaliagkas, Kallirhoe Kalinderi, Patroklos Vareltzis, Despoina Moraitou, Theodora Papamitsou, Maria Chatzidimitriou

**Affiliations:** 1 Department of Biomedical Sciences, School of Health Sciences, International Hellenic University, Alexandrion University Campus, 57400 Sindos, Greece; chdimitr@ihu.gr; 2 Laboratory of Medical Biology-Genetics, School of Medicine, Faculty of Health Sciences, Aristotle University of Thessaloniki, 54124 Thessaloniki, Greece; kkalinde@auth.gr; 3 Department of Chemical Engineering, School of Engineering, Aristotle University of Thessaloniki, 54124 Thessaloniki, Greece; pkvareltzis@cheng.auth.gr; 4 Laboratory of Psychology, School of Psychology, Aristotle University of Thessaloniki, 54124 Thessaloniki, Greece; despoinamorait@gmail.com; 5 Histology and Embryology Department, Faculty of Medicine, Aristotle University of Thessaloniki, 54124 Thessaloniki, Greece; thpapami@auth.gr

**Keywords:** Abeta, tau, CSF, Alzheimer’s disease

## Abstract

Alzheimer’s disease (AD) is a rapidly growing disease that affects millions of people worldwide, therefore there is an urgent need for its early diagnosis and treatment. A huge amount of research studies are performed on possible accurate and reliable diagnostic biomarkers of AD. Due to its direct contact with extracellular space of the brain, cerebrospinal fluid (CSF) is the most useful biological fluid reflecting molecular events in the brain. Proteins and molecules that reflect the pathogenesis of the disease, e.g., neurodegeneration, accumulation of Abeta, hyperphosphorylation of tau protein and apoptosis may be used as biomarkers. The aim of the current manuscript is to present the most commonly used CSF biomarkers for AD as well as novel biomarkers. Three CSF biomarkers, namely total tau, phospho-tau and Abeta42, are believed to have the highest diagnostic accuracy for early AD diagnosis and the ability to predict AD development in mild cognitive impairment (MCI) patients. Moreover, other biomarkers such as soluble amyloid precursor protein (APP), apoptotic proteins, secretases and inflammatory and oxidation markers are believed to have increased future prospects.

## 1. Introduction

Alzheimer’s disease (AD) is a chronic progressive neurodegenerative disease that is the most common form of dementia (affecting about 60–70% of all dementia cases). The new Diagnostic and Statistical Manual of Mental Disorders (DSM-V) diagnostic criteria have replaced the term “dementia” with major neurocognitive disorder. Unfortunately, its prevalence has dramatically risen, affecting about 55 million people worldwide, a number that is expected to double every 20 years, especially in developing countries [1]. The lifetime risk for AD is estimated to be about 10.5% for a 65-year-old person, with prevalence doubling every 5 years, reaching nearly 50% by age 80. The ε4 allele of the apolipoprotein E (APOE) gene is a strong genetic risk factor for AD development and carriage of one ε4 allele is believed to speed up the symptomatic onset of AD by about 10 years [2].

Definite diagnosis of AD is only obtained at autopsy via the histological quantification of two AD hallmarks: brain amyloid plaques (that consist primarily of amyloid-β (Aβ) peptide that is produced from the proteolytic processing of a transmembrane protein, amyloid precursor protein (APP), by β- and γ-secretases, and intraneuronal neurofibrillary tangles, that consist of tau protein, a microtubule-associated protein that is found predominantly in neurons and is responsible for the stability and assembly of microtubules [3]. In AD, tau is hyperphosphorylated and as a result dissociated from microtubules and polymerized into paired helical filaments. Otherwise, for the possible or probable type of AD, diagnosis is performed with a certain percentage of error, which is increased in the early stages of disease, when the symptoms are unclear and difficult to interpret [1].

The use of technologies for support and care for people with AD is growing. Several technological solutions are proposed with the most prominent being monitoring the patient’s vital signs and health condition changes with the help of wearable devices. Smart home technologies may help dementia patients live more independently in their homes for a longer time.

Mild cognitive impairment (MCI) is a clinical entity that has attracted clinical and research interest in recent years, mostly due to the fact that MCI patients are at increased risk for developing AD, which might range from 10 to 30% per year [4]. MCI includes impairment in both memory and nonmemory cognitive domains, therefore MCI patients can be classified into two groups: amnestic and nonamnestic. This classification by subtype might possibly predict the type of dementia that MCI patients may develop. The pathophysiological changes observed in MCI are intermediate between normal aging and dementia. Cholinergic deficit was also observed in the amnestic form of MCI due to neuronal loss in the basal nucleus of Meynert. It is believed that the early identification of patients who will convert to AD may offer the opportunity of therapeutic intervention in the initial stages of the neuropathologic processes leading to AD, thereby substantially increasing the probability of therapeutic success. However, in some cases, due mostly to nonpharmacological interventions, not all MCI patients progress to AD, and some cases even return to normal aging.

Due to its direct contact with extracellular space of the brain, cerebrospinal fluid (CSF) reflects molecular events in the brain and is an ideal candidate to identify potential AD biomarkers such as proteins and molecules that reflect the pathogenesis of the disease, e.g., neurodegeneration, inflammation and apoptosis (Table 1). That is why they are preferred over blood-based biomarkers in AD to reflect brain pathophysiology. CSF plays the role of a “brain cushion” providing basic mechanical brain protection inside the skull and it can be obtained via lumbar puncture. Although lumbar puncture is an invasive method it is a low-cost, well-tolerated and highly accessible test compared to the image-based methods that are used in AD diagnosis such as brain MRI.

A lot of progress has been made over recent years to improve AD diagnosis, resulting in biomarker-based research diagnostic criteria [5]. The core CSF biomarkers for AD pathophysiology are CSF Abeta42, t-tau and p-tau that are believed to have the highest diagnostic accuracy for early AD diagnosis [6,7,8,9,10]. These accurate biomarkers also allow the detection of preclinical AD, in the absence of evident clinical symptoms. The presymptomatic detection of AD is very important, as it can aid in the initiation of a rapid and effective treatment for the disease. Moreover, other biomarkers such as apoptosis markers, soluble APP, inflammatory and oxidation markers and secretases are studied and believed to have increased future prospects. This review will cover the most common CSF biomarkers that are used in AD diagnosis as well as some novel biomarkers that have been recently proposed and could be used as accurate diagnostic and prognostic biomarkers of AD.

## 2. Results

### 2.1. Routinely Used Biomarkers

#### 2.1.1. Beta-Amyloid (1-42) (Abeta42)

**Figure 1:**
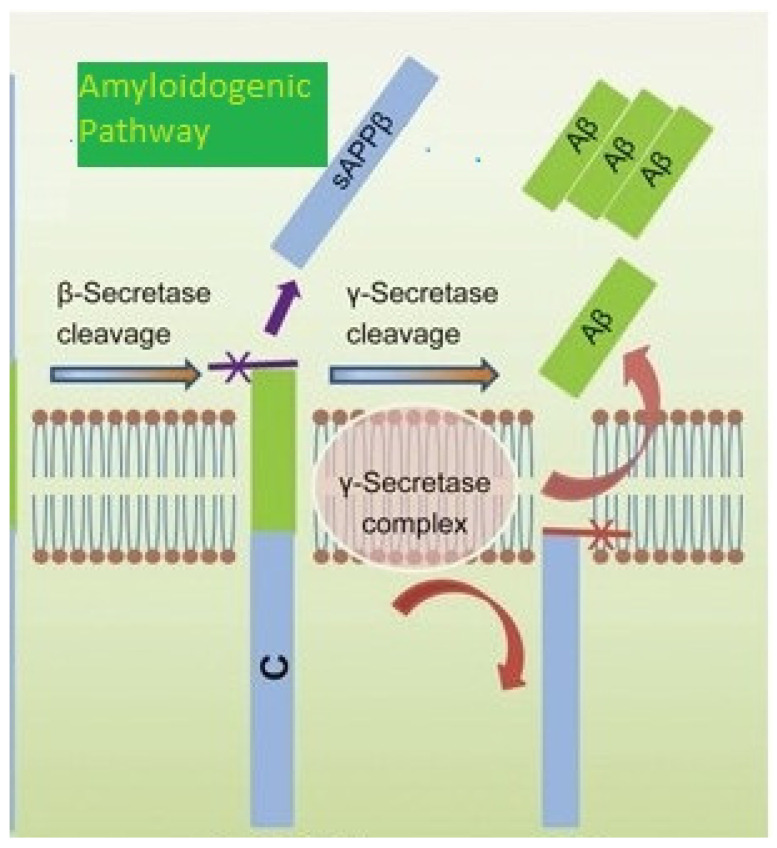
APP Processing in Alzheimer’s Disease.

Abeta42 is a 42 amino acid peptide that is the main constituent of amyloid plaques, one of the hallmarks of AD. It is derived from the proteolytic cleavage of amyloid precursor protein (APP) with the help of certain enzymes, β- and γ-secretases, that results in two major Aβ isoforms: Aβ42 (42 residues long) and Aβ40 (40 residues long) (Figure 1). Aβ1-42 is the main component of senile plaques. 

CSF Abeta42 levels are significantly reduced in AD patients compared to normal controls [11,12,13] due to its increased deposition in the amyloid plaques and a significant correlation has been found between increased amounts of amyloid plaques in the cortex and hippocampus and reduced CSF Abeta42 levels [14,15]. Reduced CSF levels of Abeta42 were also observed in other dementia types, such as Lewy body disease [16,17], Creutzfeldt–Jakob disease [18], vascular dementia and frontotemporal dementia [19]. However, these findings may be related to mixed pathology with dual pathology and overlap of the above-mentioned diseases. No significant correlation has been found between the duration of the disease and the patients’ MMSE score [20]. Mattsson et al. [21], who studied CSF biomarkers in MCI patients, found that CSF Abeta42 compared to t-tau and p-tau is the most accurate biomarker for the diagnosis of the MCI patients that will convert to AD with 79% sensitivity and 65% specificity. This agrees with van Harten et al. [22], who suggested that Abeta42 is the most accurate predictor of clinical progression in patients with subjective cognitive complaints, and a more recent study by Ewers et al. [23], who found that CSF Abeta42 showed the best diagnostic accuracy among CSF biomarkers. 

Contrary to most research studies, Jensen et al. [24] observed increased CSF Abeta42 levels in MCI patients compared to controls, but there were no follow-up studies on the MCI patients that converted to AD. 

On the other hand, in other studies, no significant difference was observed in the total CSF Abeta levels in AD patients and healthy controls [25,26], therefore the levels of total Abeta were not considered an accurate AD diagnostic marker.

CSF Abeta42 was also found in the preclinical asymptomatic phase of AD and levels in the lower part of the reference range are strongly associated with future Aβ positivity [27]. In normal subjects, high correlation was observed between blood and CSF Abeta42 levels, whereas no such correlation was observed in MCI and AD patients [28], which is believed to be due to the reduction of CSF Abeta42 levels. A similar study was performed on CSF and blood Abeta42 levels [29], which found no difference in the plasma levels of Abeta40 and Abeta42 in MCI patients that developed AD compared to stable MCI patients and healthy controls. On the other hand, CSF Abeta42 levels were reduced in MCI patients compared to controls. This difference was attributed by the authors to the fact that there is no correlation between blood and CSF Abeta42 levels. Although plasma Abeta42 levels cannot yet be considered as an independent AD marker, their changes might reflect AD conversion [30]. However, blood Abeta42 levels are not considered to be reliable compared to CSF Abeta42. CSF Abeta oligomers correlated with cognitive decline in MCI and AD patients [31] and, in particular, Abeta40 oligomers were also proposed as a potential biomarker in AD [32]. Moreover, Aβ isoforms (Aβ1-37 and Aβ1-38) may help to differentiate AD from frontotemporal and Lewy body dementia [33].

#### 2.1.2. Total Tau Protein (t-Tau)

Tau protein is the basic element of the neurofibrillary tangles (all six isoforms are located in these tangles) and is located mainly in the neuraxons, then dendrites and the cell bodies [34], therefore CSF t-tau shows the extent of neuronal damage and neurodegeneration in AD.

Increased CSF levels of t-tau were observed in MCI patients [11,35] and AD patients [12,13,14] compared to normal subjects. The increase in t-tau levels can be detected from the very early stages of AD and is stable as time goes by [12]. Moreover, it has great sensitivity and specificity in the differential diagnosis of AD from normal aging (93% and 86%, respectively) and depression, where the levels are normal [12]. In recent studies [36,37], elevated levels of t-tau and p-tau were observed in MCI patients that developed AD compared to stable MCI patients and normal control subjects. On the other hand, in stable MCI patients, no significant difference was observed in the protein levels between the exams and compared to healthy control subjects in the time period of 2 years [36]. Ivanoiu et al. [38] studied MCI patients and observed that t-tau levels were correlated more with memory function, whereas Abeta42 levels were correlated with the stage of the disease.

Although it seems to be a reliable diagnostic tool in the differential diagnosis between MCI, AD and normal subjects, an increase in t-tau levels is not considered an accurate diagnostic marker for AD, because it is also increased in other neurodegenerative diseases [39,40]. In particular, the aggregates of tau protein are the main features of several other tauopathies, including frontotemporal dementia, progressive supranuclear palsy and corticobasal degeneration. Increased levels of CSF tau protein were also observed in ischemic stroke [41] and trauma. 

t-Tau levels are very increased in Creutzfeldt–Jakob disease [42], slightly to moderately increased in neurodegenerative diseases such as AD and normal in diseases without neurodegeneration [43]. Moreover, t-tau and p-tau may be used for differential diagnosis of MCI from other diseases such as major depression [44]. A correlation between the CSF levels of t-tau and newly used techniques for AD diagnosis was sought. According to Toolboom et al. [45], the positive association between (18)F-FDDNP and CSF t-tau suggested that a part of the specific signal of (18)F-FDDNP in AD patients is due to tangle formation. A similar positive relationship between CSF tau/p-tau (181) and the amount of cortical amyloid was also observed [46]. 

#### 2.1.3. Phosphorylated t-Protein (p-Tau)

**Figure 2:**
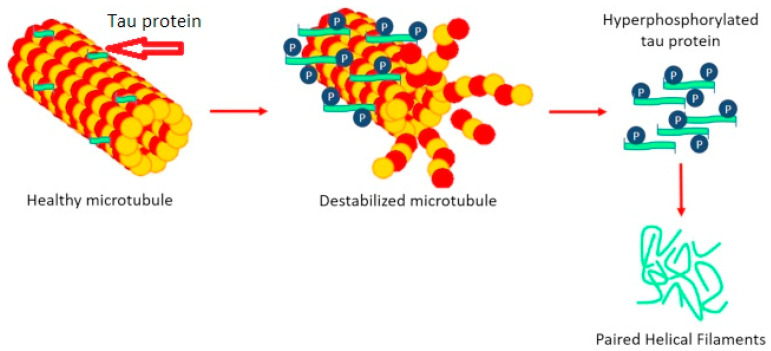
Phosphorylation of Tau protein. The red arrow shows how Tau protein stabilizes microtubules.

The abnormal phosphorylation of tau protein has toxic actions and negatively regulates its ability to stimulate microtubule assembly, leading to detachment of tau from microtubules and its accumulation into neurofibrillary tangles (Figure 2).

It is caused, to an extent, by the loss of balance between the activities of t-kinase and t-phosphatase [47,48]. Several forms of p-tau such as 396/404 [49], phosphorylated tau protein in threonin 181 [50] and phosphorylated tau protein in threonin-231 [51] were studied with an ELISA method. Ishiguro et al. [52] observed that p-tau protein levels were significantly higher in AD patients compared to healthy subjects and the discrimination between the two groups was more accurate using p-tau than t-tau. Therefore, increased levels of p-tau are considered a more reliable marker in AD early diagnosis. p-Tau is not a simple marker of axonal damage like t-tau, but is more closely related to AD pathophysiology and NFT formation. Therefore, the combination of the two tau forms is more accurate in AD diagnosis. Together with isoprostane and the volume of the hippocampus, they are useful markers in the differential diagnosis of MCI patients from healthy subjects, whereas the increase in p-tau levels and the reduction in Abeta levels are correlated with volume loss in the hippocampus [53]. In particular, p-tau in threonin-231 (P-tau231) is considered to be the most accurate marker of MCI conversion to AD [54] with 80% specificity and 80% total diagnostic accuracy in distinguishing the MCI patients that will later convert to AD. Recent studies have demonstrated that it increases early in the development of AD pathophysiology [55]. Other p-tau epitopes phosphorylated at other sites such as p-tau217 and p-tau231 have emerged over recent years to be at least as useful as p-tau231.

### 2.2. Combination of Biomarkers

The combination of CSF Abeta42, t-tau and p-tau levels has 95% sensitivity and 83% specificity in determining the patients that will develop AD [26]. Similar results were observed by Andreasen et al. [56] and Hampel et al. [57] who observed total diagnostic accuracy of over 80%. Increased levels of CSF t-tau and p-tau together with decreased levels of CSF Abeta42 create a typical AD biomarker profile and are found in the majority of AD patients. The combination of CSF proteins together with other diagnostic procedures can increase the accuracy of AD diagnosis. Low CSF Abeta42 and high t-tau levels combined with medial temporal atrophy are associated with increased risk of MCI conversion to dementia, mostly AD. In particular, the diagnostic value of CSF markers was 3-fold that of the medial temporal lobe alone [58]. 

Biomarker ratios were also studied and were frequently found to be superior compared to their individual constituents. The ratio of CSF t-tau/Abeta42 protein shows promising results in predicting future MCI [59] and dementia [60] in cognitively normal older adults. Moreover, the sensitivity and specificity values of this ratio for AD diagnosis are 85.7% and 84.6%, respectively [61]. 

CSF Aβ40/Aβ42 and CSF Aβ42/Aβ40 ratios increase the differential diagnosis of AD from other dementia types such as frontotemporal dementia, vascular dementia and Lewy body dementia. They were found to be significantly lower compared to other dementias [62]. In large-scale MCI studies [55,63], a CSF AD profile for t-tau and Abeta42 was proposed with a high accuracy of AD prediction in amnestic MCI patients (OR 26.8, 95% CI 1.6–456.4). Models that can accurately predict AD diagnosis based on CSF Abeta42 and p-tau were proposed [64,65]. 

Several studies have suggested the combination of CSF proteins and event-related potential (ERP) waveforms as an accurate diagnostic marker of MCI conversion to AD [66,67,68]. The combination of CSF and MRI markers was also studied with an accuracy of 91.8% in distinguishing between AD patients and controls. The combination was better than using either CSF or MRI biomarkers alone [68]. A low variability was observed in the CSF biomarkers during the day in AD patients and, consequently, continuous CSF measurement is considered accurate [69]. Therefore, combinations of CSF biomarkers can discriminate AD patients from normal subjects better than if they are used individually. 

### 2.3. Novel Biomarkers

#### 2.3.1. Beta-Site Amyloid Precursor Protein Cleaving Enzyme (BACE1)

Secretases are proteolytic enzymes that cleave amyloid precursor protein (APP) to form Abeta. CSF BACE1 levels were significantly elevated in MCI and AD patients compared to controls [70,71,72], AD-like biomarker profile patients [73] and in MCI patients compared to controls [74,75]. Moreover, CSF BACE1 activity was found to be increased in ApoE4 carriers compared to ApoE4 noncarriers in both MCI and AD patients, showing an association between ApoE4 genotype and BACE1 activity [75]. On the other hand, in a more recent study no difference was observed in CSF BACE1 levels between AD, MCI patients and controls [76]. CSF BACE1 levels were associated with hippocampal atrophy in AD patients [77]. Plasma secretase activity was proposed as a potential biomarker in AD [78]. However, in the ADNI cohort study, it was observed that neither CSF BACE1 levels nor sAβPPβ concentrations could be used to discriminate between healthy elderly and AD individuals [79].

#### 2.3.2. Inflammation Markers

The role of inflammation in AD is highly supported; therefore, CSF markers of inflammation such as interleukins were also studied as AD biomarkers with inconsistent results. Several studies found that interleukin-6 was increased in AD patients compared to healthy controls [80,81,82], whereas others found no statistical difference [82,83,84,85]. On the other hand, no significant difference was observed in interleukin-1 levels [86] and other interleukins such as IL-12 and IL-10 [87]. Other inflammatory markers such as tumor necrosis factor-α (TNF-α) have also been studied with contradictory results: increased [88] or no difference between AD patients and controls.

Chitinase-3-like protein 1 (YKL-40) is a marker of glial inflammation in AD that was found to be increased in AD patients compared to controls, indicating that it might be helpful as an inflammatory biomarker in AD patients [89,90]. When used together with CSF Abeta42 levels, it might be used as a biomarker for preclinical AD [91]. The CSF ratio of YKL-40/Aβ42 was found be predictive of the onset of cognitive symptoms.

#### 2.3.3. Neurogranin

Neurogranin is a postsynaptic protein that plays a role in synaptic activity and plasticity and long-term potentiation (LTP) by regulating calcium-mediated signaling pathways. Neurogranin is closely related to synaptic loss in AD patients and is expressed mainly in brain regions that are affected in AD such as cortex, hippocampus, and amygdala. CSF neurogranin levels are increased in MCI and AD patients compared to controls. Furthermore, increased CSF neurogranin is specific to AD among the various neurodegenerative disorders [91,92].

#### 2.3.4. Soluble APP (sAPP)

Low α- and β-cleaved soluble APP (sAPPα and sAPPβ, respectively) were observed in AD patients, especially those in advanced clinical stage, suggesting that these markers might be related to the severity of the disease [93]. Soluble APP was also studied in MCI patients and it was observed that sAPPβ was higher in MCI patients that converted to AD compared with stable MCI patients, suggesting it may be a more accurate biomarker in AD diagnosis than Abeta42 [94,95]. According to the results of a current multicenter study [96], sAPPα and sAPPβ are promising biomarkers in separating AD from other types of dementias with high sensitivity and specificity. On the other hand, in early studies, no difference was observed in sAPPα and sAPPβ levels [97] between AD patients and controls.

#### 2.3.5. Neurotrophic Factors

Cholinergic neurons in the brain require NGF for maintenance of cholinergic phenotype, are critical for cognition and are affected in the early stages of AD. NGF metabolism is impaired in AD patients, however, it is not known how it is correlated with AD pathology and clinical symptomology. CSF nerve growth factor (NGF) levels were significantly increased in AD patients compared to controls [98].

BDNF, a protein whose main role is to support the survival of existing neurons and encourage the growth and differentiation of new neurons and synapses, was found to be associated with AD [99,100]. However, further CSF studies indicated that it could be not taken as a marker of the disease [101,102], because it might also be increased in other neuropsychiatric disorders [101].

### 2.4. Oxidative Stress

Oxidative stress is a biological mechanism that is driven by the imbalance between reactive oxygen species (ROS) and antioxidative defense mechanisms. It is involved in the pathophysiology of MCI and AD by leading to the formation of amyloid plaques and neurofibrillary tangles. Several proteins, such A1AT, 1 α-1B-glycoprotein, serotransferrin, APOE, gelsolin, PGDS and DBP, were observed to be increasingly oxidized in MCI and AD patients compared to controls.

### 2.5. Other Biomarkers

Cystatin C is believed to protect nerve cells against Abeta-induced toxicity [103,104]. Lower CSF cystatin C levels were observed in AD patients compared to controls and correlated positively with Abeta42, Abeta40 and tau levels [105,106]. Visinin-like protein-1 (VILIP-1), a marker of neuronal injury, was also studied and higher CSF levels were observed in AD patients compared to controls, suggesting its possible role as a prognostic AD marker [107,108]. The ubiquitin proteasome system (UPS) is involved in the pathophysiology of AD and, in particular, ubiquitin and ubiquitin carboxyl-terminal hydrolase L1 (UCH-L1) are found in amyloid plaques and neurofibrillary tangles in AD patients. CSF UCH-L1 levels were elevated in AD patients compared to controls, thus suggesting its possible use as a CSF biomarker [109].

### 2.6. Neurofilament Light Chain

Neurofilament light chain (NfL) is a neuronal cytoplasmic protein that forms part of the cytoskeleton and has gained attention over recent years for the diagnosis of neurodegenerative diseases, including AD. It is used as a marker of neuronal degeneration and is associated with hippocampal atrophy and gray matter. NfL is released in the extracellular space and is extremely sensitive to AD onset and progression. CSF levels were significantly higher in AD patients compared to controls and might be used as a diagnostic and prognostic marker [110,111]. Moreover CSF NfL levels were increased in cognitively normal subjects with evidence of tau pathology and neurodegeneration, indicating that they might be used as an early prognostic biomarker of neurodegeneration and synaptic dysfunction [112]. However, the specificity of increased CSF NfL for AD is debatable as CSF NfL levels are also increased in other neurodegenerative diseases [113,114]. Moreover, Mattson et al. observed no correlation between CSF NfL and CSF Aβ42, indicating that NfL may suggest neurodegeneration independently of Aβ pathology [115]. A recent study has observed that CSF NfL levels were more closely correlated with tau protein than Abeta [116]. Recent advances in the area of proteomics offer the potential to search for novel CSF biomarkers by using modern methods.

## 3. Discussion

Despite the huge investment over recent years in AD drugs, there is currently no effective disease-modifying treatment. The first new treatment for over 20 years, aducanumab, has encountered great controversy and was not granted approval by the European authorities. One of the major concerns and research targets nowadays in the fight against AD is the finding of accurate biomarkers that will enable early diagnosis and, as a result, prompt AD treatment. Moreover, these biomarkers might be used in the evaluation of therapeutic efficacy of several disease-modifying treatments that are currently under way. In order for a biomarker to be validated for use in clinical practice, it must be able to reliably reflect disease pathogenesis. According to the results of the above-mentioned studies, the diagnostic sensitivity and specificity of CSF biomarkers in differentiating AD from MCI and healthy controls, as well as other types of dementia, seem to be achieved with promising results. Moreover, a combination of multiple robust biomarkers such as CSF Abeta42, CSF t-tau and CSF p-tau is believed to increase diagnostic and prognostic accuracy for AD. Measurements of CSF Abeta42 and p-tau protein levels are already included in recent research diagnostic criteria for AD [117,118], however, they should be further improved by novel CSF biomarkers. Therefore, CSF biomarkers have clinical utility in the differentiation between AD, MCI and normal subjects, and further discovery and validation are essential in order to improve early AD diagnosis and accelerate the development of new therapies.

When compared with novel AD biomarkers such as amyloid-PET that can detect the presence of amyloid-β plaques, CSF seems to be less accurate for AD diagnosis. On the other hand, amyloid-PET is considered to be an expensive procedure and is not widely available, whereas lumbar puncture is safe and cost-effective [119]. Moreover, blood-based biomarkers have had great prospects over recent years due to the ease and low cost of ample acquisition and analysis and have been proposed as potential accurate AD diagnostic biomarkers [120].

The study has several limitations. Firstly, this is a literature rather than systematic review, therefore it does not provide a systematic search process for inclusion and exclusion of the articles. The interpretation of the study findings is limited by the small sample sizes and high methodological heterogeneity that was observed in several studies.

Moreover, no studies that confirmed Alzheimer’s disease diagnosis with postmortem brain study findings were found.

## 4. Conclusions

CSF biomarkers (in particular, Abeta42 and tau protein as well as t-Tau/Abeta42 ratio) can be used in routine clinical practice in distinguishing AD from controls, as well as other types of dementia.

## 5. Future Directions

Although CSF biomarkers are highly accurate and sensitive, their use in clinical practice is limited compared to other biomarkers. The main reason behind this is because lumbar puncture is an invasive method for obtaining the CSF sample and there are inconsistencies in CSF sample analysis. Future directions that are commonly suggested in the field of diagnostic biomarkers is the need to implement standardized cut-off values as well as methodological consistency that will apply across all laboratories worldwide (i.e., CSF sample collection, transportation, storage). There is a need for better understanding of which biomarkers, either individually or in combination with other biological, neuroimaging or neurophysiological biomarkers, can be used for accurate early diagnosis and prognosis of AD. By addressing these knowledge gaps, it is possible that diagnostic CSF biomarker profiles will be developed for MCI, AD and other dementias that will provide great benefit in the prognosis and diagnosis of neurocognitive disorders.

## Figures and Tables

**Table 1:** List of common AD biomarkers according to disease pathophysiology.

Disease Pathophysiology	Biomarkers
Abeta deposition	Abeta isoforms (1-37, 1-38, 1-40, 1-42) Soluble APP Secretases
Neurofibrillary tangle formation	P-tau_231_, P-tau_181_
Neuronal degeneration	T-tau NfL VILIP-1
Inflammation	Interleukins-2, -6, -10 TNF YKL-40
Oxidative stress	A1AT, 1 α-1B-glycoprotein, serotransferrin, APOE, gelsolin, PGDS and DBP
Apoptosis	Cytochrome c Calpain
Neuroprotection	BDF, NGF, cystatin C

## Data Availability

Data is contained within the article.
